# Risk-Predictive and Diagnostic Biomarkers for Colorectal Cancer; a Systematic Review of Studies Using Pre-Diagnostic Blood Samples Collected in Prospective Cohorts and Screening Settings

**DOI:** 10.3390/cancers13174406

**Published:** 2021-08-31

**Authors:** Sophia Harlid, Marc J. Gunter, Bethany Van Guelpen

**Affiliations:** 1 Department of Radiation Sciences, Oncology, Umeå University, 90187 Umeå, Sweden; sophia.harlid@umu.se; 2 Nutrition and Metabolism Branch, International Agency for Research on Cancer, 69372 Lyon, France; gunterm@iarc.fr; 3 Wallenberg Centre for Molecular Medicine, Umeå University, 90187 Umeå, Sweden

**Keywords:** colorectal neoplasms, cancer screening tests, biomarkers, liquid biopsy, early detection of cancer, precision medicine

## Abstract

**Simple Summary:**

Currently, colorectal cancer screening typically involves stool tests, but a blood test might be more acceptable for screening participants. Most research on blood biomarkers for colorectal cancer has been conducted using samples from patients and may not be as predictive for early-stage cancer or pre-cancerous tumors. This systematic review summarizes the evidence from studies that used samples collected before the onset of symptoms. The quality of the studies was generally high, but very few potential biomarkers showed consistent, clinically relevant results across more than one study. Of these, the anti-p53 antibody was the most promising marker. Panels of biomarkers performed better than single markers. The results of this review underscore the need for validation of promising colorectal cancer biomarkers in independent pre-diagnostic settings.

**Abstract:**

This systematic review summarizes the evidence for blood-based colorectal cancer biomarkers from studies conducted in pre-diagnostic, asymptomatic settings. Of 1372 studies initially identified, the final selection included 30 studies from prospective cohorts and 23 studies from general screening settings. Overall, the investigations had high quality but considerable variability in data analysis and presentation of results, and few biomarkers demonstrated a clinically relevant discriminatory ability. One of the most promising biomarkers was the anti-p53 antibody, with consistent findings in one screening cohort and in the 3–4 years prior to diagnosis in two prospective cohort studies. Proteins were the most common type of biomarker assessed, particularly carcinoembryonic antigen (CEA) and C-reactive protein (CRP), with modest results. Other potentially promising biomarkers included proteins, such as AREG, MIC-1/GDF15, LRG1 and FGF-21, metabolites and/or metabolite profiles, non-coding RNAs and DNA methylation, as well as re-purposed routine lab tests, such as ferritin and the triglyceride–glucose index. Biomarker panels generally achieved higher discriminatory performance than single markers. In conclusion, this systematic review highlighted anti-p53 antibodies as a promising blood-based biomarker for use in colorectal cancer screening panels, together with other specific proteins. It also underscores the need for validation of promising biomarkers in independent pre-diagnostic settings.

## 1. Introduction

Colorectal cancer is the second most common cancer in men and women globally [1], affecting roughly one in twenty people over the course of their lifetime. Largely a disease of older age, colorectal cancer incidence rates can be expected to rise as life expectancy increases in a population, but trends have been reversed in some countries, including the United States [2], largely due to the implementation of age-based general screening programs [3].

The gold standard for colorectal cancer screening is full colonoscopy. In addition to providing the best chance of detecting colorectal cancer through, ideally, inspection of the entire colorectal epithelium, colonoscopy has important advantages as a screening technique. In particular, diagnostic biopsies can be taken directly from tumors found, and many precancerous lesions can be removed. Screening colonoscopy is, therefore, a tool not only for early detection of asymptomatic colorectal cancer, but also for primary prevention. However, the implementation of colonoscopy for general screening is limited by several factors. The procedure is resource demanding, dependent on qualified personnel, uncomfortable for the patient and entails a small, but non-negligible, risk of complications such as bleeding or intestinal perforation. Achieving adequate uptake is a challenge, which is further hampered by inabilities to adequately capture all socioeconomic and ethnic groups [4].

Many countries have implemented fecal blood testing into colorectal cancer screening programs, using guaiac-based fecal occult blood tests (gFOBT) or, increasingly, quantitative fecal immunochemical tests (FIT). FIT can also be supplemented with a multitargeted tumor DNA test (FIT-DNA) that is approved for use in the United States [5]. Fecal blood testing is generally followed by sigmoidoscopy in patients with a positive result and sometime extended to full colonoscopy upon detection of polyps. Whereas full colonoscopy is effective at 10-year intervals, fecal testing is generally performed every one to three years. Moreover, limiting to sigmoidoscopy misses the roughly one third or more of colorectal cancer occurring in the proximal colon, which is more common at higher ages and in women [6,7]. 

In order to optimize colorectal cancer screening, there is a need for continued improvement of testing methods with respect to acceptability (i.e., less invasive tests), accessibility (i.e., lower costs and staffing demands) and performance. Blood-based biomarkers represent an enticing avenue toward achieving these goals. To date, one blood test, entailing measurement of methylated *Septin 9* gene (*mSEPT9*) in plasma, has been approved for colorectal cancer screening in some regions including the United States (in people who decline other screening methods). Although the discriminatory performance of *mSEPT9* is lower than for other screening methods currently in use [8,9], this may be compensated by an increased willingness of potential screening participants to undergo phlebotomy compared to stool testing or colonoscopy. The discriminatory ability of biomarkers is typically evaluated using measures such as sensitivity, specificity and receiver-operating characteristics (ROC) probability curves, in which the false positive rate is plotted on the x-axis against the true positive rate on the y-axis. The area under the ROC curve (AUC, or AUROC) ranges from 0.5, indicative of no power to separate cases from non-cases, to 1, indicative of perfect discrimination. To be clinically meaningful, biomarkers should have an AUC value as close to 1 as possible. There are no pre-defined performance thresholds for screening tests; the accuracy of novel biomarkers is generally evaluated in comparison with existing, approved tests. For FIT, the most recent systematic review from the US Preventive Services Task Force reported a pooled sensitivity of 0.74 for colorectal cancer and 0.23 for advanced adenoma, both with a specificity of approximately 0.95 [10], though the discriminatory performance varies depending on setting, test and cut-off.

Blood-based testing has several potential uses, not only as diagnostic biomarkers to help select people most likely to benefit from endoscopy and avoid unnecessary endoscopy in general screening programs, but also for risk stratification to help refine and individualize screening recommendations [11]. Risk-predictive biomarkers, in contrast to diagnostic biomarkers, would not necessarily indicate the presence of a tumor, but rather the risk of colorectal cancer over a longer time period. Such a test could be used, for example, at younger ages (e.g., 30–45 years, prior to the typical screening start at 50–60 years), to help decide when a person should enter a general screening program and perhaps what modality and frequency of screening would be most appropriate. Although colorectal cancer at ages under 50 years is rare, rates are increasing, especially for rectal cancer, and younger age groups are therefore an emerging target population for risk stratification and precision screening [12]. Risk-prediction algorithms using age, family history of cancer, genetic risk variants and lifestyle-related factors show some promise for colorectal cancer risk stratification [13,14,15,16], but have not achieved sufficient performance to guide precision screening. Novel blood-based biomarkers could, therefore, have clinical value for both risk prediction and diagnosis of colorectal cancer.

Research into blood-based biomarkers for colorectal cancer has expanded rapidly in recent years, as summarized in recent reviews [17,18]. The types of biomarkers assessed vary widely, and some of the most promising findings have been based on tumor DNA [19,20], either genetic or epigenetic. Other types of biomarkers, such as proteins, microRNA, antibodies and metabolites have also been reported to distinguish between colorectal cancer patients and controls. However, the bulk of research to date has used samples collected from patients diagnosed in clinical settings. Although such biomarkers could be very valuable for disease monitoring, their ability to detect colorectal cancer may not apply in the asymptomatic, pre-diagnostic period targeted by general screening. Studies conducted to identify and/or validate biomarkers in settings directly relevant for colorectal cancer screening, i.e., true screening settings or prospective cohorts, may be particularly valuable for the translation of findings from observational research to randomized trials and, ultimately, to clinical implementation.

The aim of this systematic review was to summarize the evidence for blood-based risk-predictive and diagnostic biomarkers of colorectal cancer identified in studies using pre-diagnostic samples from asymptomatic individuals, i.e., samples collected in prospective cohorts or general screening settings. Overall, few biomarkers demonstrated a clinically relevant discriminatory ability, especially with consistent results in more than one study. Proteins were the most common type of marker investigated, whereas markers including anti-p53 antibodies and DNA methylation at specific sites showed more consistent and stronger results, respectively. Multi-marker panels generally achieved higher discriminatory performance than single markers.

## 2. Materials and Methods

### 2.1. Eligibility Criteria

We included original, peer-reviewed, human studies presented in English and published in the past 10 years, i.e., between 1 January 2011 and 4 February 2021. Under these conditions, short reports, null results in brief and letters could be considered eligible, whereas pre-prints and conference abstracts were ineligible. The time period was chosen to balance a broad search intent with a manageable return of papers to assess for inclusion. In line with the intent of the review, only studies of blood-based biomarkers, analyzed in pre-diagnostic samples, i.e., collected in prospective cohorts or general screening settings, for the purpose of risk prediction or early diagnosis of colorectal cancer were considered eligible. Given the importance of precancerous lesions in colorectal cancer, studies including colorectal adenoma were included. Survival and therapeutic response outcomes were ineligible. We set a generous, arbitrary lower limit for sample size of 25 study subjects in at least one relevant endpoint group and in the comparison (control) group. Hereditary colorectal cancer, such as hereditary non-polyposis colorectal cancer or familial adenomatous polyposis, was an exclusion criterion, as were non-general screening settings including high-risk groups, such as familial cancer, inflammatory bowel disease and surveillance due to previous adenoma.

### 2.2. Information Sources

Searches were carried out in PubMed on 4 February 2021 and, with a modified search string, on 9 February 2021. Review articles, the reference lists of papers found in the searches, the article collections of the authors as well as post hoc searches of PubMed were used to identify additional studies not captured by the original search strings. 

### 2.3. Search Strategy

The initial search string run was: 

“(Marker OR Biomarker) AND (Serum OR Plasma OR Blood OR Circulating) AND (Diagnosis OR Screening OR “Early Detection of Cancer” [Mesh]) AND (Prospective OR “Prediagnostic” OR “prediagnostic” OR “Pre-diagnostic” OR “pre-diagnostic”) AND (Colorectal OR Colon OR Rectal) AND (Cancer OR Adenocarcinoma OR Carcinoma OR Adenoma)”.

In an informal quality check using the authors’ collections, we found that the search string missed relevant papers lacking the prospective/pre-diagnostic term. Adding the word “screening” to the term resolved the issue, but returned a dramatically higher number of hits. Therefore, we used both search strings, but for the second string including the word “screening”, we filtered the search to title and abstract only: 

“(Marker [Title/Abstract] OR Biomarker [Title/Abstract]) AND (Serum [Title/Abstract] OR Plasma [Title/Abstract] OR Blood OR Circulating [Title/Abstract]) AND (Diagnosis [Title/Abstract] OR Screening [Title/Abstract] OR “Early Detection of Cancer” [Mesh]) AND (Screening [Title/Abstract] OR Prospective [Title/Abstract] OR “Prediagnostic” [Title/Abstract] OR “prediagnostic” [Title/Abstract] OR “Pre-diagnostic” [Title/Abstract] OR “pre-diagnostic” [Title/Abstract]) AND (Colorectal [Title/Abstract] OR Colon [Title/Abstract] OR Rectal [Title/Abstract]) AND (Cancer [Title/Abstract] OR Adenocarcinoma [Title/Abstract] OR Carcinoma [Title/Abstract] OR Adenoma [Title/Abstract]).”

### 2.4. Selection Process

**Figure 1:**
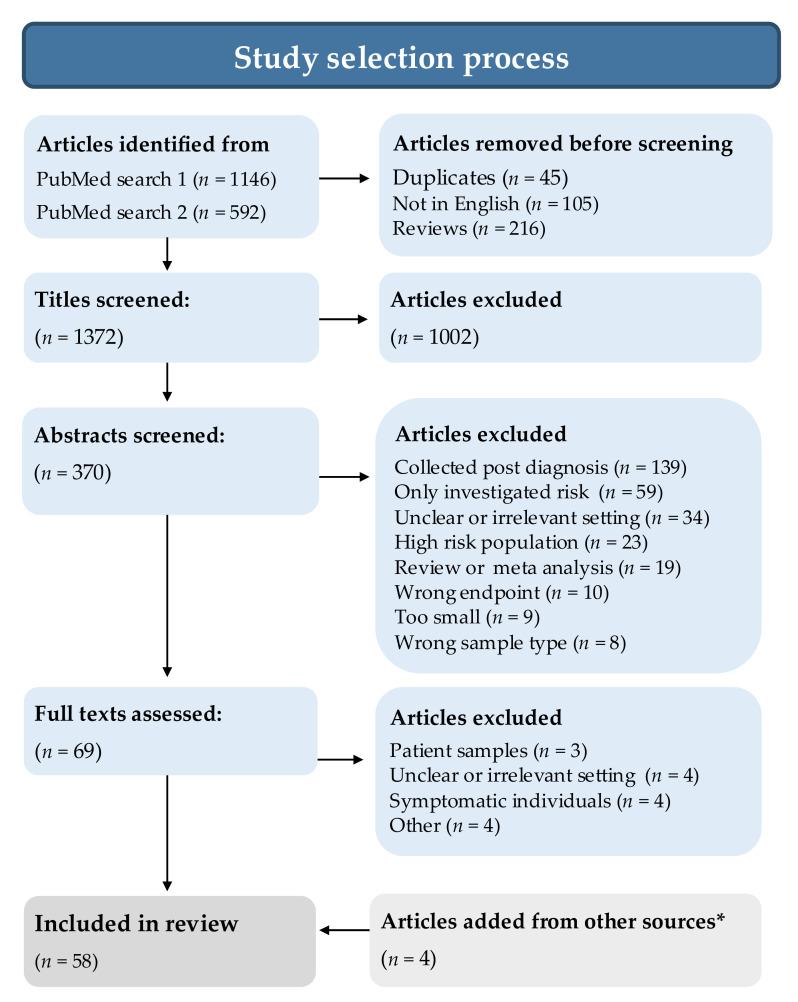
Flow diagram illustrating the selection of studies assessing blood-based risk-predictive and diagnostic biomarkers of colorectal cancer using pre-diagnostic samples from asymptomatic individuals, i.e., samples collected in prospective cohorts or general screening settings. During data extraction, an additional five studies were excluded due to lack of key information (*n* = 1), too small sample size (*n* = 2) or non-general screening population (*n* = 2). * Other sources included reference lists, review articles, the article collections of the authors and post hoc searches of PubMed.

The study selection process is summarized in Figure 1. Two of the authors (S.H. and B.V.G.) perused all study titles independently of each other and marked clearly ineligible studies for exclusion, due to obviously wrong endpoint (e.g., wrong disease, response to therapy), obviously post-diagnostic samples, samples that were not blood or studies that were completely off topic. Articles with congruent exclusion decisions were excluded. Articles with incongruent assessments or congruent short-list assessments underwent abstract examination. The same two authors then read the abstracts of all remaining studies and provided comments on why a study should be excluded. Studies with incongruent abstract assessments were discussed, and in some cases, the methods section of the full paper was checked, and if agreement was not immediately reached, we erred on the side of shortlisting for examination of the full paper. All remaining papers were read in full by either S.H. or B.V.G., and papers with uncertainties were read by all authors to reach a consensus decision. Additional studies fulfilling the inclusion criteria were identified from reference lists, reviews, article collections of the authors and post hoc searches of PubMed.

### 2.5. Data Collection Process

For data extraction and presentation, studies were classified according to setting, either prospective cohort (hereafter sometimes referred to simply as prospective) or general screening, from which data were extracted by S.H. and B.V.G, respectively. Additionally, the extracted data for a random selection of approximately 10% of the studies from both settings were checked by M.G., with no corrections.

### 2.6. Data Items

Data on study design, numbers of study participants, sample medium, biomarker analyses and main findings were tabulated, separately for prospective cohort and screening studies. Effect measures extracted were limited to area under the receiver operating curve (AUC), sensitivity and specificity (highest specificity presented in the study) and estimates of risk (odds ratio (OR), hazard ratio (HR), relative risk (RR)). Results for colorectal cancer and advanced adenoma endpoints were prioritized in the summary of main findings. For studies including multiple data sets, e.g., discovery, internal validation and/or external validation, results were extracted only for data sets meeting the criteria for inclusion in the systematic review.

### 2.7. Quality Assessment

In order to provide an overview of study quality, we used the Newcastle–Ottawa Scale (NOS) for assessing the quality of non-randomized studies in meta-analyses [21], which we adapted for biomarker studies in prospective cohorts (including nested case-control studies) and screening settings. Categories and rating scales, i.e., maximum numbers of stars per category, were retained. In the case-control scale, high-quality record linkage or registry data for case definition were considered adequate. For ascertainment of exposure in both the case-control and cohort scales, we made an overall assessment of sample handling and analytical method quality. For the comparability of cases and controls, age was considered the most important factor, whereas other factors could include sex, follow-up time from sampling to diagnosis, lifestyle factors, etc. The systematic review was registered in PROSPERO (registration number CRD42021236073), including a brief presentation of the review process.

## 3. Results and Interpretation

### 3.1. Study Selection

Searches yielded 1146 and 592 hits for search string 1 and 2, respectively. After exclusions for duplicates, articles not in English and review articles, 1372 articles remained. Based on a generous assessment of their titles (excluding only articles that were deemed clearly irrelevant by both S.H. and B.V.G.), 370 were selected for abstract screening. Among the screened abstracts, 69 were selected for full article assessment. The most common reason for excluding an abstract was the use of post-diagnostic blood samples (*n* = 139). In addition, several studies used an approach focused on risk and etiology, with no clear intent to identify or validate biomarkers from the perspective of risk prediction or early detection (*n* = 59) or had an unclear or irrelevant study setting (*n* = 34), which was confirmed by a specific check of the methods section of the full paper when the independent author assessments were incongruent. Interrater congruence was 84% at the title stage and 88% at the abstract stage. Four additional articles were identified from other sources, including reference lists, reviews, the article collections of the authors and post hoc searches of PubMed, for a total of 58 studies selected for data extraction (Figure 1).

### 3.2. Study Characteristics

We identified 31 studies conducted in a prospective cohort setting, of which one was excluded due to lack of key information (number of cases and outcome measure) [22]. Of the remaining 30 studies fulfilling the inclusion criteria, one [23] investigated adenoma and the remainder colorectal cancer. The majority of the prospective studies (*n* = 23) utilized a nested case-control design, and a few used a cohort or case-cohort design. The number of cases ranged from 32 to 4210 cases, rarely more than 500.

A total of 27 studies included data potentially stemming from a general screening setting, of which four were excluded due to small sample size or non-general screening [24,25,26,27]. Of the remaining 23 studies, 17 presented results for colorectal cancer, and 15 presented results for advanced adenoma. Case-control design was most common, of which six studies used matched controls and the remainder unmatched. Two studies used a cohort design. The number of colorectal cancer cases ranged from 25 to 59, and the number of advanced adenoma cases ranged from 37 to 420, generally fewer than 150. Half of the screening studies were based on the BliTz (Begleitende Evaluierung innovativer Testverfahren zur Darmkrebsfrüherkennung) study, a well-characterized cohort in southern Germany.

For both the prospective and screening studies, plasma or serum were the most common sample media. A few studies used other media such as extracted nucleic acids or circulating white blood cells. Most prospective studies reported either OR, AUC or both, whereas in the screening studies AUC, sensitivity and specificity were most common. 

The quality of the studies included in the review was evaluated using an adaptation of the NOS score. All studies scored high, in part because studies with a weak design, or performed in an unclear setting, were excluded at an earlier stage of the article selection process. Some studies received a lower score on selection due to lack of information on how the cases were ascertained, what the matching factors were and, for the prospective cohort studies, whether the controls were free from cancer at the start of the study.

### 3.3. Biomarkers

#### 3.3.1. Proteins

Protein markers represented the most common target in both the prospective cohort-based studies (11 of 30 selected studies) and in the screening studies (11 of 23 selected studies) (Table 1). The most frequently evaluated protein was carcinoembryonic antigen (CEA), which was included in six studies [28,29,30,31,32,33]. CEA is a known marker of colorectal cancer progression, used for surveillance of colorectal cancer patients [34]. As a single marker, CEA performed modestly well when colorectal cancer was the outcome, with AUCs ranging from 0.59 in samples collected prospectively between 3–4 years before diagnosis [31] to 0.63 for samples collected in a screening setting [30]. It performed best when included in multi-marker panels; for example, CEA combined with p53 auto antibodies yielded an AUC of 0.85 for colorectal cancer from samples collected in a screening setting [33]. However, for detecting adenomas, the highest reported AUC for panels including CEA was 0.56, which indicates that it does not have sufficient discriminatory ability to be useful for early detection or risk stratification.

Other proteins identified as potentially promising colorectal cancer biomarkers were proteins known to be either directly involved in or strongly associated with inflammation. They included C-reactive protein (CRP) (five studies [23,30,35,36,37]), macrophage chemoattractant protein-1 (MCP-1) (two studies [36,38]), interleukin-6 (IL-6) (two studies [23,28]), macrophage inhibitory cytokine 1, also known as growth differentiation factor 15, (MIC-1/GDF15) (three studies, [23,28,39]), amphiregulin (AREG) (three studies [28,39,40]) and Leucine-rich alpha-2-glycoprotein-1 (LRG1) (two studies [29,41]). The inflammatory protein included in most studies was CRP, a common marker of acute inflammation [42]. However, CRP failed to reach the top markers in two of the five studies that included it [23,36]; did not detect advanced adenoma in Tao et al., with an AUC of 0.5 [37]; and performed only modestly as an early detection biomarker in the remaining two studies, with an AUC of 0.64 for advanced adenoma in Lim et al. [30], and a combined AUC (CRP+SAA) of 0.62 for colorectal cancer in Toriola et al. [35].

Of the remaining inflammatory proteins, those that showed the strongest potential as future colorectal cancer biomarkers were AREG, LRG1 and potentially MIC-1/GDF15. AREG performed well on its own, with an AUC consistently above 0.6 [28,39,40]. When included in a multi-marker panel (that also included MIC-1/GDF15), the AUC was above 0.8 for colorectal cancer and reached 0.6 for advanced adenomas in two of the three studies [39,40]. However, AREG was evaluated as part of three screening studies that all stemmed from the BliTz screening trial. Therefore, before any general conclusions can be drawn about its performance, it would need to be evaluated in samples collected in an independent setting.

LRG1, on the other hand, was included as a marker in one prospective study based on samples from the Women’s Health Initiative (WHI) cohort [29], and in a screening study from 2020 [41]. In the case-control setting, using samples collected at least 3 months before colorectal cancer diagnosis, a panel containing LRG1 reached an AUC of 0.72. In the screening setting, a multi-marker panel containing HP, LRG1 and PON3 had an AUC of 0.65 for detection of advanced adenomas and another panel, optimized for detecting colorectal cancer, reached an AUC of 0.79.

MIC-1/GDF15 was evaluated as part of several larger panels without reaching the top-ranked markers. It was included as a separate marker for adenomas in Song et al. [23], where it performed reasonably well with an OR of 1.55 (95% CI: 1.03–2.32), AUC not shown. It is noteworthy that MIC-1/GDF15 had also been favorably evaluated in a study of recurrent adenoma, not included in the systematic review selection [43]. 

Some studies assessed candidate protein biomarkers that have shown promise in clinical colonoscopy settings. One such example is CYFRA 21-1 (cytokeratin 19 fragment) [44,45,46], which showed an AUC of 0.73 for detecting advanced adenomas in a general cancer screening setting [30]. CYFRA 21-1 was also selected for inclusion in at least one multi-marker panel tested in a screening setting [32], whereas in a prospective cohort setting, it was deemed unsuitable as a screening marker [31].

Several studies used a proteomics approach, measuring large panels of proteins in their first phases (sometimes a clinical or patient setting) and typically proceeding with validation of top hits. For example, one prospective study [47] nested within the North Sweden Health and Disease Study and five studies from the BliTz screening cohort [28,38,39,40,48] used Proseek Multiplex immunoassays (Olink Proteomics, Uppsala, Sweden), though most top hits differed. Many of the highest ranked proteins were only included in one study, making it difficult to assess reproducibility. One notable exception was fibroblast growth factor 21 (FGF-21), elevated levels of which were associated with a higher risk of colorectal cancer in the Swedish study, in which both the discovery and validation phases used pre-diagnostic samples [47], and in the BliTz validation set [48]. However, the discriminatory ability of FGF 21-1 in both studies was insufficient for clinical implementation.

#### 3.3.2. Metabolites

The metabolome was evaluated in four of the cohort-based studies [55,56,57,58] and one of the screening studies [59] included in this review (Table 2). Methods and materials differed between studies and included both liquid and gas chromatography, as well as both plasma and serum samples. One of the earlier prospective studies used a combination of methods to measure 676 metabolites in serum samples from 254 case-control pairs collected at a median of approximately 8 years prior to diagnosis [55]. Of 447 metabolites successfully identified, none were significantly associated with colorectal cancer risk. In contrast, an AUC of 0.81 was reported for a panel of 14 metabolites identified in serum by gas chromatography in a study of 31 advanced adenoma patients and 254 healthy controls from a screening program, using an approach with a training and test set [59].

A prospective study based on samples from two Shanghai cohorts, the Shanghai Women’s Health Study (SWHS) and the Shanghai Men’s Health Study (SMHS), identified metabolites using both Gas Chromatography-Time of Flight Mass Spectrometry (GC-TOFMS) and Ultra-performance Liquid Chromatography and Quadrupole Time-of-flight Tandem Mass Spectrometry (UPLC-QTOFMS) for global metabolic profiling of plasma samples from 250 case-control pairs [58]. In that study, 35 metabolites were significantly associated with colorectal cancer, nine of which retained significance after multivariable adjustments. A panel containing the top nine produced an AUC of 0.76.

The two remaining metabolomics studies both used samples collected as part of the EPIC cohort. One was a case-cohort study from EPIC-Heidelberg [56], and the other was a nested case-control study with samples from EPIC-Turin [57]. The EPIC-Heidelberg study, which included 163 colorectal cancer cases and a subcohort of 774 participants [56], analyzed levels of 120 metabolites in plasma, of which one (lysophosphatidylcholine 18:0) was inversely associated, and another (phosphatidylcholine PC ae C30:0) was positively associated, with colorectal cancer risk. Both metabolic markers may be more likely to function as risk factors rather than early disease biomarkers. The study from EPIC-Turin analyzed serum from 66 case-control pairs [57], using an untargeted metabolomics approach focused on lipophilic molecules, and identified nine features that they deemed to be of further future interest. 

Important to note is that although some metabolomics studies yielded high enough AUCs to be clinically useful, there is a lack of replication of individual metabolites. Until more studies evaluating the same targets are produced, metabolomic markers are more likely to contribute to the understanding of colorectal cancer etiology, rather than be used as biomarkers for risk prediction and early diagnosis.

#### 3.3.3. Antibodies

Among the studies selected for this review, seven included evaluations of antibodies [33,39,60,61,62,63,64], five evaluated antibodies only (listed in Table 3) and two evaluated combinations of protein panels and antibodies (listed in Table 1). A majority of the studies analyzed auto-antibodies to p53. Antibodies towards this tumor suppressor have lately attained increasing interest as a promising early detection biomarker for colorectal cancer. In the studies included in this review, two evaluated the independent association between levels of p53 autoantibodies and colorectal cancer risk [61,64], whereas an additional three included it in a multi-marker panel [33,39,62]. Teras et al. used a nested case control design with 392 cases and 774 controls drawn from the Cancer Prevention Study-II Nutrition Cohort. They found significant associations for the full case set, which were strengthened when limiting the analysis to participants diagnosed within 3 years of blood draw (RR = 2.26, 95% CI = 1.06–4.83). This time dependency was corroborated by Butt et al. in 2020 [61], using a much larger dataset including 3702 colorectal cancer cases and an equal number of controls. When stratified by follow-up time, the association in this study was significant only among cases diagnosed within 4 years of blood draw, with similar risk estimates to those presented in Teras et al. (OR = 2.27, 95% CI = 1.62–3.19).

#### 3.3.4. Nucleic Acids

In the category nucleic acids, we included all studies evaluating non-coding RNAs (five in total, [65,66,67,68,69]), as well as studies evaluating DNA markers, such as DNA methylation [8,70,71,72,73,74], mitochondrial DNA [75] and circulating tumor DNA [19] (Table 4).

Among the non-coding RNA studies, microRNAs have been most extensively investigated, but few have produced significant results. The earliest study identified in our search used a TaqMan microRNA array, as well as an in-depth literature search, to identify 12 potential microRNA targets [65], none of which reached significance in validation tests including samples from adenoma patients. Of the four studies of microRNAs, three used samples collected in screening settings [65,67,69] and one [68] used prospective samples from the Northern Sweden Health and Disease Study. In the prospective study, 12 candidate microRNAs were measured in plasma samples collected at both pre- and post-diagnostic time points from the same patients, with the top four giving an AUC for colorectal cancer detection of 0.93. However, only one (miR-21) showed a time trajectory consistent with potential use as an early detection marker for colorectal cancer, elevated approximately three years prior to diagnosis. The other two microRNA studies [67,69] both used an approach including FIT-positive and unselected patients from general screening. Using a multi-marker microRNA panel containing six markers Marcuello et al. reached an AUC of 0.80, while Zanuttoa et al., using a similarly sized panel with different microRNAs, observed an AUC of 0.61, in both studies for the detection of advanced adenomas.

One recent cohort-based study [66] analyzed a PIWI interacting RNA (piR-54265) in serum samples from 307 colorectal cancer cases and 614 matched controls from the prospective cohort study of Dongfeng-Tongji (DFTJ) in China. They found it to be significantly associated with colorectal cancer risk, primarily in individuals diagnosed within 2–3 years after blood draw. For other non-coding RNA studies included in this review, independent validation of results is lacking.

Among DNA-based markers, the most well studied is DNA methylation of *Septin 9*, with somewhat mixed results [76]. Our search identified two studies that included *SEPT9* methylation, both based on samples collected at screening [8,74] and both published before 2015. More recent studies on DNA methylation included one that evaluated genome-wide DNA methylation in leukocytes and identified three CpG sites (cg04036920, cg14472551 and cg12459502) that together produced a c-statistic of 0.74 [72]. Another DNA methylation study specifically evaluated methylation in four genes (*SFRP1*, *SFRP2*, *SDC2* and *PRIMA1*) [70], with an AUC of 0.93 for the multi-marker panel for detecting adenoma. DNA methylation in circulating tumor DNA was also the focus of a recent study using a newly constructed panel (PanSeer) with the ability to detect multiple different cancer types [19]. For colorectal cancer, a pre-diagnostic sensitivity of 94.9% was reported for samples collected up to four years before diagnosis.

#### 3.3.5. Other Markers

Aside from the types of markers already described, which were included in multiple studies, some types of biomarkers were only included in single studies (Table 5). One example is a recent investigation of the triglyceride–glucose index (TyG index) published in 2020 [79]. This easily accessible marker gave an AUC of 0.69, which is not as high as some biomarkers but would be much easier to implement. Another example of re-purposing of routine lab tests is the iron-storage protein and inflammatory marker ferritin, which was included in a promising multi-marker panel [33].

All markers, including top findings, are presented in Table 1, Table 2, Table 3, Table 4 and Table 5.

## 4. Discussion

### 4.1. Limitations of the Evidence

The investigations identified in this review were generally of high quality but varied considerably with respect to data analysis and presentation of results, and few biomarkers demonstrated a consistent, clinically relevant discriminatory ability across more than one study. As expected, the performance of the biomarkers summarized in this review was generally not sufficient for clinical implementation. The ideal circulating biomarker for screening would be released from the tumor into the bloodstream in sufficient quantities to achieve high discriminatory ability. Colorectal tumors present in asymptomatic people, particularly if they are early-stage carcinoma or advanced adenoma, may not release adequate amounts for detection, even as technological advances achieve increasingly high sensitivity. Perhaps even more importantly, not all tumors are likely to possess a given biomarker, such as a specific genetic or epigenetic alteration, or produce a specific marker protein. Testing a panel including different types of biomarkers could help overcome this limitation, as exemplified by studies including panels with both proteins and p53 autoantibodies [33,39].

Some studies presented results stratified for early- and late-stage colorectal cancer. Since detection of early-stage colorectal cancer is a premise of effective colorectal cancer screening, such analyses are highly relevant, particularly for studies conducted in a screening setting. Stage-specific results were not presented in the results tables in this review, mainly because of the generally small subgroup sizes. Colorectal cancer screening also targets the detection and removal of advanced adenoma. Of the studies included in this review, a majority of those with samples collected in general screening settings presented results for advanced adenoma as an endpoint. In contrast, only one of the studies conducted in a prospective cohort setting investigated advanced adenoma [23]. In general, findings for precancerous lesions were weak to null, with some exceptions, such as in Marcuello et al. [67], in which a panel of six microRNAs reached an AUC of 0.80 for detecting advanced adenomas in FIT+ participants in a screening setting.

A major challenge in biomarker discovery is the risk of over-fitting and chance findings. At the very least, bootstrapping, cross-validation, consideration of multiple testing and/or other statistical methods to reduce the risk of false positive findings should be applied, which was not always conducted rigorously in the studies identified for this review. Validation of discovery-stage findings is also a critical step in biomarker development, though not always possible within the same study setting as the discovery analyses. For example, the rarity of colorectal cancer events in general screening programs typically prevents division into separate discovery and validation sets. This issue can be addressed through collaborative efforts, as in most of the BliTz studies included in this review, for example, by joining forces with clinical cohorts. However, few to no biomarkers have a demonstrated clinically relevant discriminatory ability in more than one pre-diagnostic data set.

An advantage of studies set in prospective cohorts is the opportunity to address the temporality of biomarker performance. A biomarker that becomes detectable or demonstrates altered levels close to diagnosis would be a strong candidate for a screening test to supplement or replace fecal testing, whereas a biomarker that differentiates future cases from controls but without a clear time trajectory would more likely be a biomarker of risk. The latter could still have relevance for screening, primarily to improve risk-prediction algorithms to inform precision screening protocols with respect to starting age and screening frequency.

In order to distinguish between potential risk-predictive and diagnostic biomarkers, repeated pre-diagnostic samples represent a particularly valuable resource. We previously used such a design in a validation study inspired by promising findings from the Alpha-Tocopherol, Beta-Carotene (ATBC) cohort for the gut hormone ghrelin [85]. Murphy et al. observed dramatically higher colorectal cancer risk in ATBC participants with low circulating total ghrelin concentrations in samples collected within 10 years prior to case diagnosis (OR: 10.86, 95% CI 5.01 to 23.55), whereas an inverse association was observed at longer follow-up times. This relationship was not replicated in our analysis of a unique set of 60 matched case-control pairs with repeated, pre-diagnostic plasma samples (one sample collected within 5 years prior to case diagnosis and one sample collected 10 years earlier), despite adequate statistical power [86]. There was no obvious explanation for the diverging findings, which demonstrates the value of validation studies in observational settings prior to clinical testing.

A major disadvantage of prospective cohorts for cancer biomarker research is the inherently limited sample volumes available for analysis. Whereas plasma/serum volumes of several milliliters are typically required for analyses of circulating tumor DNA, especially for asymptomatic patients with low tumor burden, analyses in biobank samples must usually be limited to sample volumes of 500 μL or less.

### 4.2. Limitations of Review Processes

A major limitation of the review process was the use of general search strings for a broad topic, which included many different types of exposures. Studies using the specific name of the biochemical analyte or platform, without referring to them as biomarkers or markers, would be missed in our searches. Furthermore, search string 2 could potentially miss relevant research published in a form with no abstract, such as a short report or letter. The aforementioned ghrelin publication by the authors was missed for this reason [86].

We also found it difficult to establish defined criteria to distinguish between studies focusing on etiology versus studies aimed towards identifying suitable biomarkers for screening. This problem was especially prominent for the prospective cohort studies. Although biomarkers investigated to help elucidate etiological mechanisms could certainly have relevance as biomarkers for screening, we recognize that the studies identified in our searches represent a minute fraction of all such publications. Therefore, we only included studies for which risk prediction or early diagnosis was clearly in focus, for example, as a specified aim or with calculation of discriminatory ability. Although this is in line with the stated purpose of this review, it was not noted specifically as a restriction in the PROSPERO registration.

Limiting the review to papers published from 2011 and onward may have led to relevant studies being missed. We accepted this risk based on the consideration that important novel biomarkers identified more than 10 years ago would likely have been validated in studies during the past 10 years. Our eligibility criteria will also have missed any promising biomarkers published only in non-English papers.

In order to assess the quality of the studies included in this review, we applied the Newcastle–Ottawa Scale (NOS) for assessing the quality of non-randomized studies in meta-analyses. We adapted the scale for use in assessing biomarker studies, making an effort to minimize the modifications. This may have introduced a bias toward higher scores, particularly with respect to the scoring category for exposure in the case-control scale. For example, using the same method of exposure ascertainment for cases and controls is standard procedure in this type of biomarker study design. However, the generally high scores noted also reflect the inclusion criteria for the review, which were set to ensure selection of studies with sampling prior to diagnosis, i.e., high-quality study designs. Most studies also accounted for factors such as age, typically by matching of cases and controls or by multivariable adjustment, and were thus awarded two NOS stars for the category on comparability of cases and controls. However, for cancer screening, the practice of matching controls has been called into question [87], and some studies, therefore, made an active decision not to use matched controls [28,39,53].

### 4.3. Implications for Practice and Policy

Taken together, this systematic review did not identify any single biomarker or biomarker panel that consistently demonstrated a discriminatory ability on par with FIT, suggesting that stool testing in general colorectal cancer screening is unlikely to be replaced by a blood test in the foreseeable future. Though not accurate enough to be used alone, autoantibodies to p53 showed consistently promising results as a marker for early diagnosis of colorectal cancer and might serve as a supplement to methylated *Septin 9* testing or in a future multi-marker panel. In general, panels of biomarkers performed better than single markers. The results of this review underscore the need for validation of promising colorectal cancer biomarkers in independent pre-diagnostic settings prior to clinical testing and implementation.

Translation of biomarkers to clinical implementation requires consideration of factors beyond discriminatory ability. The optimal biomarker would be insensitive to variable pre-analytical conditions, such as time of day for sample collection, fasting status and sample handling. It would be collected in standard phlebotomy tubes and be analyzed on equipment available at larger hospital laboratories. Many of the more promising biomarkers in this review, including anti-p53 antibodies, could be developed to fulfill these considerations. However, these are not absolute requirements for a clinical blood test. For example, interleukins degrade rapidly at room temperature, but IL-6 is routinely analyzed in clinical practice. Metabolites are often sensitive to fasting status [88], which could be a disadvantage if samples are to be used in biomarker panels for risk stratification, but a metabolomics-based diagnostic biomarker reflecting the tumor itself might be less likely to be affected by food intake. The results of a biomarker test should also be easy to interpret, which does not exclude the possibility of multi-marker or omics-based tests requiring advanced data analyses to generate results. The explosion of genomic and transcriptomic tumor testing in recent years, such as FoundationOne and PAM50, and the rapid implementation in clinical oncology practice, illustrate the willingness of clinicians to adopt and familiarize themselves with modern, data-heavy analyses.

A health-economical evaluation is central to the implementation of any medical testing, including cancer screening. Demonstrating cost effectiveness for a colorectal cancer screening test with a discriminatory performance on par with current fecal testing alternatives should not be difficult given the high and increasing costs of therapy, as the drug arsenal expands and survival during therapy continues to improve. However, for a test with a high positivity rate, cost effectiveness approaching that of colonoscopy screening might be achieved simply by chance, i.e., by the high proportion of screening participants selected for colonoscopy. This issue has been raised for annual *SEPT9* testing, which would send 70% of screenees to colonoscopy within 5 years [89]. Conversely, the potential of a highly discriminatory biomarker test to reduce unnecessary colonoscopy, beneficial from both a patient and health-economy perspective, should not be overlooked. Risk stratification, using prediction algorithms, potentially supplemented with biomarkers, might not only be helpful to select and encourage high-risk individuals to attend earlier or more frequent screening, but also to identify very low-risk individuals who might safely postpone their screening start.

Risk-prediction and diagnostic biomarkers could also have value in the clinical setting, to help shorten the time to diagnosis in patients with symptoms potentially consistent with a colorectal tumor but otherwise low suspicion of malignancy. Such an aid to clinical decision could be implemented in referral guidelines [90], similar to the recent addition of FIT to the NICE guidelines for example [91]. From a secondary prevention perspective, effective and personalized risk stratification could help guide surveillance strategies after adenoma removal.

**Figure 2:**
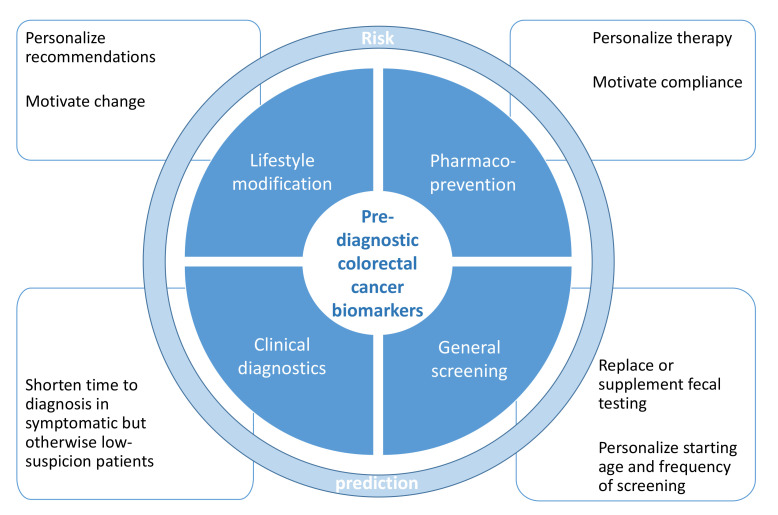
Potential applications of pre-diagnostic biomarkers for prevention and early detection of colorectal cancer.

In addition, there are other potential preventative benefits of blood-based biomarkers (Figure 2). The minimally invasive nature of blood testing should be conducive not only to improving overall screening uptake, but ideally also to reducing socioeconomic disparities in participation rates. A biomarker panel indicative of risk over a longer time period could be used for precision lifestyle counselling and/or pharmacoprevention, especially if it could detect specific negative physiological effects of poor lifestyle behaviors or metabolic health. Candidate pharmacopreventive drugs exist, for example the antidiabetic drug Metformin and aspirin and other non-steroidal anti-inflammatory drugs [92,93,94] and a targeted approach might improve both compliance and numbers needed to treat/harm.

### 4.4. Future Research Perspectives

The numbers of studies using pre-diagnostic blood samples to investigate colorectal cancer biomarker is limited compared to the overwhelming volume of publications based on patient samples. In part, this is likely due to the relatively large volumes of blood required for some types of biomarkers, such as tumor DNA-based markers and extra-cellular vesicles (especially in asymptomatic tumor bearers), rendering such analyses generally unfeasible in prospective cohort biobanks. Prospective cohorts have also traditionally focused primarily on etiological biomarker studies, with the aim of elucidating how colorectal cancer arises and grows, including mechanistic links between lifestyle-based exposures and carcinogenesis. However, with the rapid expansion of large-scale proteomics and other technologies using smaller sample volumes, prospective cohorts seem poised to become a key asset for translating biomarkers to the clinic. Furthermore, novel collaborative efforts such as the international Colorectal Cancer Pooling Project (C2P2, originally planned with a risk factor and etiology focus) may prove invaluable as a resource for research of blood-based risk-predictive and diagnostic biomarkers, with large sample sizes allowing for analyses of various time points prior to diagnosis and of clinical and molecular tumor subgroups. Such resources could also provide opportunities for validation in various geographical, ethnic and socioeconomic settings. The extensive etiological biomarker data previously collected in many prospective cohorts might also be revisited to identify multi-marker panels for risk stratification, using, for example, machine learning methods.

For future studies, we would stress the importance of a clear and complete description of the samples used, in particular distinguishing between screening, clinical and mixed colonoscopy settings. In new etiological studies in prospective cohorts, scientists might consider the possible additional value of evaluating the biomarkers also from the perspective of risk prediction, with appropriate statistical analyses and lag-time stratification as pre-specified analyses. We also support standardized reporting of results according to published guidelines and checklists, such as the Standards for Reporting Diagnostic Accuracy (STARD) statement [95], and the Transparent Reporting of a multivariable prediction model for Individual Prognosis Or Diagnosis (TRIPOD) Statement [96], to aid interpretation of findings.

## 5. Conclusions

In this systematic review, we evaluated 53 articles that investigated risk-predictive or diagnostic biomarkers of colorectal cancer using blood samples collected in a pre-diagnostic, asymptomatic setting. All studies used samples collected either in prospective cohorts (months to years before diagnosis) or in general screening settings. The quality of the studies was generally high, but very few potential biomarkers showed consistent results in more than one study. The vast majority focused on protein biomarkers in plasma or serum, but even when combined into multi-marker panels, proteins alone did not achieve sufficient discriminatory ability to be clinically useful as an alternative to FIT in general colorectal cancer screening. However, one of the most promising biomarkers, p53 autoantibodies, consistently performed well, especially in combination with protein markers, which may warrant development as a supplement to current screening tests. In general, panels of biomarkers performed better than single markers.

The search for colorectal cancer biomarkers that can detect early carcinomas or advanced adenomas, or aid in the identification of high-risk individuals, has relied too heavily on samples collected from patients after diagnosis, whose tumor burden and systemic response may not be representative of the general screening setting. The findings of this review underscore the need for discovery and validation of biomarkers in independent, pre-diagnostic, asymptomatic settings, in order to improve the chances of successful translation to clinical implementation.

## Figures and Tables

**Table 1:** Studies of inflammatory markers and other proteins, sometimes supplemented by antibodies, analyzed in pre-diagnostic blood samples (collected in prospective cohort or general screening settings) for the purpose of risk prediction or early diagnosis of colorectal cancer.

Reference	Cohort (Design)	Time from Sampling to Diagnosis (Cohort Setting Only)	CRC	Adenoma	Contr./ Cohort	Biomarker/ Platform	Main Findings	Adapted NOS Scale ** Max: Selection = ★★★★ Comp. = ★★ Exp./Outc. = ★★★
**Cohort setting**								
Ladd et al. Cancer Prev. Res, 2012 [29]	WHI (Nested case control)	245 days (mean) 109 days (mean)	90 32	-	90 * 32 *	Proteomics (MS): (1) 5022 unique protein IDs (2) 1779 quantified (3) 6 significant (*p* < 0.05)	Top markers: MAPRE1, LRG1, IGFBP2, Enolase 1, ARMET, PDIA3 Panel: MAPRE1, LRG1, IGFBP2 + CEA Validation set (4 marker panel): AUC: 0.72 Sensitivity: 41% Specificity: 95%	★★★ ★★ ★★★
Touvier et al. World J Gastroentero, 2012 [36]	SUVIMAX (Nested case control)	6.5 years (median)	50	-	100 *	Proteins: hs-CRP, Adiponectin, Leptin, sVCAM-1, sICAM-1, sE-selectin, MCP-1	Top markers: Adiponectin Panel: Adiponectin, sVCAM Adiponectin: OR: 0.45 (95% CI: 0.22–0.91. *p* = 0.03) Panel (Adiponectin, sVCAM): AUC: 0.98	★★★ ★★ ★★★
Toriola et al. Int J Cancer, 2013 [35]	WHI (Nested case control)	3 years (cutoff, follow up)	988	-	988 *	CRP, SAA	CRP (5th vs. 1st quintile, colon) OR: 1.37 (95% CI: 0.95–1.97) SAA (5th vs. 1st quintile, colon) OR: 1.26 (95% CI: 0.88–1.80) AUC (Both): 0.62 (95% CI: 0.55–0.68)	★★★ ★★ ★★
Thomas et al. Brit J Cancer, 2015 [31]	UKCTOCS (Nested case control)	4 years (cutoff, serial samples)	40	-	40 *	CEA, CYFRA21-1, CA12	Top marker: CEA All stages AUC (CEA): 0–1 year before diagnosis: 0.74 1–2 years before diagnosis: 0.64 2–3 years before diagnosis: 0.61 3–4 years before diagnosis: 0.59	★★★★ ★★ ★★★
Bertuzzi et al. BMC Cancer, 2015 [49]	EPIC- FLORENCE (Nested case control)	3 years (mean)	48	-	48 *	Global proteome analysis (phase 1 + 2) Targeted proteome analysis (phase 3): APOC2, CLU, CO4-B, CO9, FETUA, MASP2, MBL2, GRP2	CLU (men only) AUC: 0.72 Sensitivity: 95% Specificity: 75%	★★★ ★★ ★★★
Song et al. Cancer Prev Res, 2016 [23]	NHS (Nested case control)	10 years (median)		757	757 *	MIC-1, CRP, IL-6, sTNFR-2	MIC-1: (5th vs. 1st quintile) OR: 1.55 (95% CI: 1.03–2.32)	★★★★ ★★ ★★★
Shao et al. Cancer Epidemiol Biomarkers Prev, 2017 [50]	AFHSC/DoDSR (Nested case control)	8 years (cutoff, serial samples)	397	-	397 *	Proteomics (MALDI-TOF MS)	Proteomic peaks: 2886.67, 2939.24, 3119.32, and 5078.81 The 4 peaks associated with CRC 1 year before diagnosis. Sensitivity: 69% Specificity: 67%	★★★★ ★★ ★★★
Song et al. Int. J Cancer, 2018 [51]	JPHC (Case- cohort)	9.5 years (median)	457	-	751	67 inflammatory and immunity markers	Top markers: CCL2/MCP1, CCL3/MIP1A, CCL15/MIP1D, CCL27/CTACK, CXCL6/GCP2, sTNFR2 HR (4th vs. 1st quartile) CCL2/MCP1: 1.69 sTNFR2: 1.61 CCL15/MIP1D: 1.39 CCL27/CTACK: 1.35 CXCL6/GCP2: 0.70 CCL3/MIP1A: 0.61 Significance lost after adjustments	★★★★ ★★ ★★★
Rho et al. Gut 2018 [52]	CHS(Nested case control)	0–1 years (31 cases) 1–3 years (35 cases)	79	-	79 *	Discovery: 1100 markers Pre validation: 78 markers	Final panel: BAG4, IL6ST, VWF, EGFR, CD44 Panel, all cancers versus all controls: AUC: 0.86 Sensitivity: 73% Specificity: 90%	★★★ ★★ ★★★
Harlid et al. Sci Rep, 2021 [47]	NSHDS (Nested case control)	0.7 years (median) 6.7 years (median)	58 450	-	58 * 450 *	Olink proteomic panels (Inflammation and Oncology II)	Top markers: FGF-21, PPY FGF-21, colon OR: 1.23 95% CI 1.03–1.47 6 marker panel, colon AUC: 0.63 PPY, rectum OR: 1.47 95% CI 1.12–1.9 AUC: 0.61	★★★★ ★★ ★★★
**Screening setting**								
Chen H et al. Clin Cancer Res, 2015 [28]	BliTz (discovery set)	-	35	-	54	PEA (Olink Oncology I), 92 proteins	Top markers: (AUC > 6): AREG, CEA, GDF-15, IL-6 Multi-marker (8 proteins): AUC 0.76 (0.65–0.85), sensitivity 44% at 90% specificity	★★★★ - ★★★
Wen Y-H et al. Clin Chim Acta, 2015 [32]	General health screening at patient’s expense, Taoyuan, Taiwan	-	26	-	See footnote ***	AFP, CA 15-3, CA 125, PSA, SCC, CEA, CA 19-9, CYFRA 21-1	Top markers: CEA, sensitivity 53.8%, CYFRA 21-1 sensitivity 38.9 Multi-marker panel (all 8 markers): sensitivity 76.9%	★★★★ - ★★★
Tao S, et al. Br J. Cancer, 2015 [37]	BliTz	-	-	AA: 193	225	CRP, sCD26, complement C3a anaphylatoxin, TIMP-1	CRP: AUC 0.50 (0.45–0.55) C3a: AUC 0.52 (0.47–0.57) sCD26: AUC 0.54 (0.49–0.59) TIMP-1: AUC 0.58 (0.53–0.63)	★★★★ ★ ★★★
Werner S, et al. Clin Cancer Res, 2016 [33]	BliTz (validation study)	-	36	AA: 420	1200	CEA, ferritin, seprase, osteopontin, anti-p53 antibody ****	5-marker panel, CRC: AUC 0.78 (0.68–0.87), sensitivity 42% (26–59) at 95% specificity 5-marker panel, AA: AUC 0.56 (0.53–0.59), sensitivity 9% (6–12) at 95% specificity CEA+anti-p53, CRC: AUC 0.85 (0.78–0.91), sensitivity 45% at 95% specificity CEA+anti-p53, AA: AUC 0.56 (0.53–0.59), sensitivity 6% at 95% specificity	★★★★ - ★★★
Butt J et al. Int J Cancer, 2017 [53]	BliTz	-	50	AA: 100 NAA: 30	228	Multiplex serology (11 proteins) for *Streptococcus gallolyticus* subsp. *gallolyticus* Tested: individual proteins, any protein, ≥2 of 6-protein panel, Gallo2178-Gallo217 double-positivity	CRC: Gallo2178: OR 3.19 (1.11–9.21) AA: Gallo0933: OR 2.02 (CI: 1.01–4.04)	★★★★ ★★ ★★★
Chen H et al. Clin Epidemiol, 2017 [39]	BliTz (validation set)	-	41	AA: 106	107	PEA (Olink Oncology I v.2, 92 proteins) and serum p53 antibodies	Top markers, CRC: 12 proteins in both discovery and validation sets using Wilcoxon (10 with AUC > 6) Multi-marker (GDF-15, AREG, FasL, Flt3L), CRC: AUC 0.81 (0.73–0.88), sensitivity 53.6% at 90% specificity, AA: AUC 0.58 (0.51–0.65), sensitivity 18.9 at 90% specificity Multi-marker + p53, CRC: AUC 0.82 (0.74–0.90), sensitivity 56.4 at 90% specificity, AA: 0.60 (0.52–0.69), sens. 22.0 at 90% specificity	★★★★ ★★ ★★★
Qian J et al. Br J Cancer 2018 [48]	BliTz (validation set)	-	45	AA: 80 NAA: 72	250 *	PEA (Olink Inflammation I, 92 proteins)	FGF-21, CRC: AUC 0.71 (0.61–0.81), sensitivity 37.1% at 90% specificity, OR highest vs. lowest tertile 3.92 (1.51–12.18) FGF-21, AA: 0.57 (0.50–0.63), sensitivity 11.1% at 90% specificity, OR highest vs. lowest tertile 2.24 (1.18–4.44)	★★★★ ★★ ★★★
Qian et al. J Clin Epidemiol, 2018 [38]	BliTz (validation set)	-	42	-	84 *	PEA (Olink Inflammation I, 92 proteins)	Individual proteins: AUC > 6 for 13 proteins, of which 5 overlapped with discovery set results. Sensitivity >25% at 90% specificity for 5 proteins, of which one overlapped with discovery results. 5-protein panel (FGF-23, CSF-1, Flt3L, DNER, MCP-1): AUC 0.59 (0.47–0.70), sensitivity 28.6% and 11.9% at 90% and 95% specificity, respectively	★★★★ ★★ ★★★
Lim DH, et al. J Clin Lab Anal, 2018 [30]	Screening patients, Cheonan, South Korea	-	-	AA: 59 NAA: 232	223	CYFRA 21- 1, CEA, CA19- 9, AFP, hsCRP	Top markers, AA: CYFRA 21-1: AUC 0.732 (0.656–0.809), sensitivity 30.5%, CEA: AUC 0.628 (0.542–0.714) sensitivity 11.8%, hsCRP: AUC 0.637 (0.559–0.715), sensitivity not presented	★★★ - ★★
Bhardwaj M et al. Cancers, 2019 [40]	BliTz (validation set)	-	56	AA: 101	102 *	PEA. Tested 12 overlapping proteins from LC/MRM-MS and PEA (Olink Oncology II, Immune response and Cardiovascular III): CDH5, Gal, IGFBP2, MASP1, MMP9, MPO, OPN, PON3, PRTN3, SPARC, TFRC (TR), AREG	Top markers, CRC (AUC > 6): CDH5, OPN, TR, AREG Multi-marker, CRC (MASP1, OPN, PON3, TR, AREG): AUC 0.82 (0.74–0.89), sensitivity 50% at 90% specificity Multi-marker, AA (MASP1, OPN, PON3, TR, AREG): AUC 0.60 (0.51–0.69)	★★★★ ★★ ★★★
Bhardwaj M et al. Eur J Cancer, 2020 [41]	BliTz (validation set)	-	56	AA: 99	99 *	LC/MRM-MS, 270 proteins	Individual markers, CRC (44 proteins): AUC range 0.53 (0.44–0.63) to 0.77 (0.69–0.84) Multi-marker, CRC (A1AT, APOA1, HP, LRG1, PON3): AUC 0.79 (0.70–0.86), sensitivity 46% at 90% specificity Multi-marker, AA (early-stage CRC panel: HP, LRG1, PON3): AUC 0.65 (0.56–0.73), sensitivity 25% at 90% specificity	★★★★ ★★ ★★★
Li B, et al. Cancer Biomarkers, 2020 [54]	Health exam project, not otherwise specified, Jiangsu, China	-	50	AA: 50	150 *	Netrin-1	CRC: OR highest vs. lowest (optimal cut-off) = 7.731 (3.618–16.519), AUC 0.759 (0.680–0.837), sensitivity 46% at 90% specificity AA: null	★★ ★★ ★★★

Abbreviations (not including biomarker names, for which the reader is referred to the original study article): **AA**, Advanced Adenoma; **AFHSC**, Armed Forces Health Surveillance Center; **AUC**. Area Under the Curve: **BliTz**, Begleitende Evaluierung innovativer Testverfahren zur Darmkrebs-Früherkennun, Germany; **CHS**, Cardiovascular Health Study; **CRC,** Colorectal Cancer: **DoDSR**, Department of Defense Serum Repository; **EPIC**, European Prospective Investigation into Cancer and Nutrition; **HR**. Hazard Ratio: **JPHC**, Japan Public Health Center-based Prospective Study; **LC/MRM-MS**, liquid chromatography/multiple reaction monitoring-mass spectrometry; **MS**, Mass Spectrometry; **NAA**, non-advanced adenoma; **NHS**, Nurses’ Health Study; **NSHDS**, North Sweden Health and Disease Study; **OR**, odds ratio; **PEA**, proximity extension assay; **SUVIMAX**, Supplémentation en VItamines et Minéraux AntioXydants; **SWHS**; Shanghai Women’s Health Study; **UKCTOCS,** UK Collaborative Trial of Ovarian Cancer Screening; **WHI**, Women’s Health Initiative. * Matched controls. ** Newcastle–Ottawa Quality Assessment Scale for case-control studies, adapted for use for assessment of biomarker studies conducted in a screening setting. For case-control studies: selection (max 4 stars), comparability (max 2 stars), exposure (max 3 stars). For cohort studies: selection (max 4 stars), comparability (max 2 stars), outcome (max 3 stars). *** Cohort design, total ***n*** = 41,516, NOS assessment using the cohort scale. **** CYFRA 21-1 from the original panel was described in the statistics section as having been found to be “dispensable” in an extra colorectal cancer enriched re-optimization study and it was therefore not included in further analyses.

**Table 2:** Studies of metabolites analyzed in pre-diagnostic blood samples (collected in prospective cohort or general screening settings) for the purpose of risk prediction or early diagnosis of colorectal cancer.

Reference	Cohort (Design)	Time from Sampling to Diagnosis (Cohort Setting Only)	CRC	Adenoma	Contr./ Cohort	Biomarker/ Platform	Main Findings	Adapted NOS Scale ** Max: Selection = ★★★★ Comp. = ★★ Exp./Outc. = ★★★
**Cohort setting**								
Kühn et al. BMC Med, 2016 [56]	EPIC-HEIDELBERG (Case-cohort)	6.6 years (median)	163	-	774	120 metabolites: (acylcarnitines, amino acids, biogenic amines, phosphatidylcholines, sphingolipids, and hexoses)	Top markers: LysoPC a C18:0, PC ae C30:0 LysoPC a C18:0 (4th vs. 1st quartile) OR: 1.84 (95% CI: 1.02–3.34) PC ae C30:0 (4th vs. 1st quartile) OR: 0.50 (95% CI: 0.28–0.90)	★★★ ★★ ★★★
Shu et al. Int J Cancer, 2018 [58]	SWHS/SMHS (Nested case control)	Time stratification: <4 years and >4 years	250	-	250 *	Metabolites in plasma: 35 metabolites associated with CRC at FDR-*p* < 0.05	Top 9 panel: AUC: 0.76 Top 2 single metabolites: Picolinic acid: OR: 5.11 (95% CI: 2.33–11.20) PE(20:0/18:2): OR: 0.45 (95% CI: 0.29–0.70)	★★★★ ★★ ★★★
Cross et al. Cancer, 2014 [55]	PLCO (Nested case control)	7.8 years (median)	254	-	254 *	676 serum metabolites (metabolon)	Leucyl-leucine (90th vs. 10th percentile)OR: 0.50 (95% CI: 0.32–0.80) Glycochenodeoxycholate (90th vs. 10th percentile, sex stratified) OR: 5.34 (95% CI: 2.09–13.68) Significance lost after adjustments	★★★★ ★★ ★★★
Perttula et al. BMC Cancer, 2018 [57]	EPIC-TURIN (Nested case control)	7.5 years (median)	66	-	66 *	Lipophilic metabolites incl. (ULCFAs): 8690 features, 9 selected	Top markers: IDs: 5080, 3207, 6054 and 839 Classification rate: 72%	★★ ★★ ★★★
**Screening setting**								
Farshidfar F et al. Br J Cancer, 2016 [59]	Screening patients, Calgary, Canada (discovery)	-	-	A: 31	254	GC-MS untargeted metabolomics	Multi-marker profile: (14 metabolites): AUC 0.81 (0.70–0.92)	★★★ ★★ ★★★

Abbreviations (not including biomarker names, for which the reader is referred to the original study article): **CI,** confidence interval, **CRC**, colorectal cancer; **EPIC**, European Prospective Investigation into Cancer and Nutrition; **GC-MS**, gas chromatography–mass spectrometry; **OR**, odds ratio; **PLCO**, Prostate, Lung, Colorectal, and Ovarian cancer screening trial; **SMHS**, Shanghai Men’s Health Study; **SWHS**, Shanghai Women’s Health Study. * Matched controls. ** Newcastle–Ottawa Quality Assessment Scale for case-control studies, adapted for use for assessment of biomarker studies conducted in a screening setting. For case-control studies: selection (max 4 stars), comparability (max 2 stars), exposure (max 3 stars). For cohort studies: selection (max 4 stars), comparability (max 2 stars), outcome (max 3 stars).

**Table 3:** Studies of antibodies analyzed in pre-diagnostic blood samples (collected in prospective cohort or general screening settings) for the purpose of risk prediction or early diagnosis of colorectal cancer.

Reference	Cohort (Design)	Time from Sampling to Diagnosis (Cohort Setting Only)	CRC	Adenoma	Contr./ Cohort	Biomarker/ Platform	Main Findings	Adapted NOS Scale ** Max: Selection = ★★★★ Comp. = ★★ Exp./Outc. = ★★★
**Cohort setting**								
Pedersen et al. Int J Cancer, 2014 [63]	UKCTOCS (Nested case control)	6.8 years (median)	97	-	94 *	Autoantibodies: MUC1, MUC2 and MUC4	Top markers: MUC1-STn, MUC1-Core3 MUC1-STn Sensitivity: 8.2% Specificity: 95% MUC1-Core3 Sensitivity: 13.4% Specificity: 95%	★★★★ ★ ★★★
Butt et al. Cancer Epidemiol Biomarkers Prev, 2018 [60]	CLUE, CPSII, HPFS, MEC, NHS, NYUWHS, PHS, PLCO, SCCS and WHI (Nested case control)	4–18 years (median, different studies)	4210	-	4210 *	Antibody responses to 9 Streptococcus gallolyticus (SGG) proteins	Top marker: Gallo2178 Gallo2178 All cases: OR: 1.23 (95% CI: 0.99–1.52) Diagnosed <10 years after blood draw: OR: 1.40 (95% CI: 1.09–1.79)	★★★ ★★ ★★★
Teras et al. Cancer Epidemiol Biomarkers Prev, 2018 [64]	CPSII (Nested case control)	11 years (follow up)	392	-	774 *	p53 autoantibodies	All cases: RR: 1.77 (95% CI: 1.12–2.78) Diagnosed <3 years after blood draw: RR: 2.26 (95% CI: 1.06–4.83)	★★★★ ★★ ★★★
Butt et al. Cancer Epidemiol Biomarkers Prev, 2020 [61]	CLUE, CPSII, HPFS, MEC, NHS, NYUWHS, PHS, PLCO, SCCS and WHI (Nested case control)	7 years (median)	3702	-	3702 *	p53 autoantibodies	All cases: OR: 1.33 (95% CI: 1.09–1.61) Diagnosed <4 years after blood draw: OR: 2.27 (95% CI: 1.62–3.19)	★★★ ★★ ★★★
Screening setting								
Chen H et al. Oncotarget, 2016 [62]	BliTz (validation study)	-	49	AA: 99 NAA: 29	100	Autoantibodies to 64 tumor associated antigens Tested: individual proteins and 2- to 6-marker panels	Top hits: TP53, anti-IMPDH2, anti-MDM2, anti-MAGEA4 Best 2-marker panel (TP53, anti-IMPDH2): sensitivity CRC 10% (4–22), sensitivity AA 7 (3–14), specificity 95 (89–98) Best 6-marker panel (TP53+IMPDH2+MDM2 +MAGEA4+CTAG1 +MTDH), Sensitivity CRC 24% (15–38), sensitivity AA 25% (18–35), specificity 85% (77–91)	★★★★ - ★★★

Abbreviations (not including biomarker names, for which the reader is referred to the original study article): **AA**, advanced adenoma; **BliTz**, Begleitende Evaluierung innovativer Testverfahren zur Darmkrebs-Früherkennun, Germany; **CLUE**, Campaign Against Cancer and Stroke; **CPSII**, Cancer Prevention Study-II; **CRC**, colorectal cancer; **EPIC**, European Prospective Investigation into Cancer and Nutrition; **HPFS**, Health Professionals Follow-up study; **MEC**, Multiethnic Cohort Study; **NAA**, non-advanced adenoma, **NHS**, Nurses’ Health Study; **NYUWHS,** NYU Women’s Health Study; **OR**, odds ratio; **PHS**, Physicians’ Health Study; **PLCO**, Prostate, Lung, Colorectal, and Ovarian Screening Study; **SCCS**, Southern Community Cohort Study; **UKCTOCS,** UK Collaborative Trial of Ovarian Cancer Screening; **WHI**, Women’s Health Initiative. * Matched controls. ** Newcastle–Ottawa Quality Assessment Scale for case-control studies, adapted for use for assessment of biomarker studies conducted in a screening setting. For case-control studies: selection (max 4 stars), comparability (max 2 stars), exposure (max 3 stars). For cohort studies: selection (max 4 stars), comparability (max 2 stars), outcome (max 3 stars).

**Table 4:** Studies of nucleic acids analyzed in pre-diagnostic blood samples (collected in prospective cohort or general screening settings) for the purpose of risk prediction or early diagnosis of colorectal cancer.

Reference	Cohort (Design)	Time from Sampling to Diagnosis (Cohort Setting Only)	CRC	Adenoma	Contr./ Cohort	Biomarker/ Platform	Main Findings	Adapted NOS Scale ** Max: Selection = ★★★★ Comp. = ★★ Exp./Outc. = ★★★
**Cohort setting**								
Wikberg et al. Cancer Med, 2018 [68]	NSHDS/VIP (Nested case control)	20 years (maximum follow up)	58	-	147 *	12 miRNAs	Top panel: miRNA-21, miR-18a, miR-22, miR-25 4 marker panel: AUC: 0.93 Sensitivity: 67% Specificity: 90%	★★★★ ★★ ★★★
Mai et al. Theranostics, 2020 [66]	DFTJ (Nested case control)	9 years (follow up)	307	-	614 *	Serum piR-54265	All cases: OR: 2.10 (95% CI: 1.66–2.65) Diagnosed <1 years after blood draw: OR: 2.80 (95% CI: 1.60–4.89) Diagnosed <2 years after blood draw: OR: 2.45 (95% CI: 1.49–4.03) Diagnosed <3 years after blood draw: OR: 1.24 (95% CI: 0.90–1.72)	★★★ ★ ★★★
Huang et al. Cancer Epidemiol Biomarkers Prev, 2014 [75]	SWHS (Nested case control)	Time stratification: <5 years and >5 years	444	-	1423	mtDNA copy number	OR (2nd vs. 3rd tertile): 1.26 (95% CI: 0.93–1.70) OR (1st vs. 3rd tertile): 1.44 (95% CI: 1.06–1.94)	★★★★ ★ ★★★
Dietmar Barth et al. J Natl Cancer Inst, 2015 [71]	EPIC-HEIDELBERG (Nested case control)	6.4 years (mean)	185	-	807	“ImmunoCRIT” Cell type specific DNA methylation in *Foxp3*, *CD3* and *GAPDH* loci	ImmunoCRIT HR (3rd vs. 1st tertile): 1.59 (95% CI: 0.99–2.54)	★★★★ ★★ ★★★
Onwuka et al. BMC Cancer, 2020 [73]	EPIC-TURIN (Nested case control)	6.2 years (mean)	166	-	424 *	Blood DNA methylation CpG- sites	Methylation risk score (MRS), based on 16 CpGs. OR (original dataset): 2.68 (95% CI: 2.13–3.38) OR (testing dataset): 2.02 (95% CI: 1.48–2.74) AUC: 0.82	★★★ ★ ★★★
Chen et al. Nat Commun, 2020 [19]	TZL (Nested case control)	4 years (cutoff, follow up)	35	-	414	PanSeer panel: Circulating tumor DNA from pre-diagnostic stomach, esophageal, colorectal, lung or liver cancer patients	Pre-diagnosis sensitivity (all cancers): 94.9 (95% CI: 88.5–98.3)	★★★★ ★★ ★★★
**Screening setting**								
Warren JD, et al. BMC Med, 2011 [74]	Screening patients, single community clinic, USA (validation)	-	-	A: 78	See footnote ***	*SEPT9* methylation, rtPCR in triplicate	Sensitivity 10%	★★★★ - ★★★
Luo X, et al., PLoS ONE, 2013 [65]	BliTz (validation set)	-	-	AA: 50	50	Five miRNAs from discovery phase (miR-29a, -106b, -133a, -342-3p, -532-3p), seven candidate miRNAs (miR-18a, -20a, -21, -92a, -143, -145, -181b)	Null	★★★★ - ★★★
Church T, et al., Gut, 2014 [8]	PRESEPT **** (validation study)	-	53	AA: 314 NAA: 209	934	*SEPT9* methylation (Epi proColon Assay)	≥1/2 runs positive, CRC: Sensitivity 48.2% (32.4–63.6), specificity 91.5% (89.7–93.1) ≥1/3 runs positive, CRC (post hoc): Sensitivity 63.9% (47.5–79.2), specificity 88.4% (86.2–90.4) ≥1/2 runs positive, AA: Sensitivity 11.2% (7.2–15.7) compared to 9.2% positive rate in controls	★★★★ ★★ ★★★
Maffei et al. Mutagenesis, 2014 [77]	FOB+ screening patients, Bologna, Italy	-	25	26 “polyps”	31	Micronucleus frequency in peripheral blood lymphocytes	Mean micronucleus frequency in CRC > polyps > controls (all 3 *t*-tests *p* < 0.001)	★★ ★★ ★★★
Heiss JA, Brenner H Clin Epigenetics, 2017 [72]	BliTz (clinical+screening for discovery, divided for modelling)	-	46	-	46 *	Leucocyte DNA methylation array	Top markers: cg04036920, cg14472551, cg12459502 Multi-marker (3 markers): C-statistic 0.74 (0.57–0.87)	★★★★ ★★ ★★★
Myint NNM, et al. Cell Death Dis, 2018 [78]	FOBT+ patients, BCSP	-	-	Pre- neoplastic lesions: 76	37	Total cfDNA, and tumor-related mutations (*BRAF*, *KRAS* by ddPCR) and patient-specific assays for trunk mutations identified by multiregional targeted NGS of adenoma tissues	Null	★★ - ★★★
Barták BK, et al. Epigenetics, 2018 [70]	Screening patients, not otherwise specified (validation study)	-	47	AA: 37	37	DNA methylation of *SFRP1*, *SFRP2*, *SDC2* and *PRIMA1*	Individual markers, CRC: all AUC >8, adenoma: all AUC > 6 Multi-marker (4 genes), CRC: AUC 0.978 (0.954–1.000), sensitivity 91.5%, specificity 97.3% Multi-marker (4 genes), adenoma: AUC 0.937 (0.885–0.989), sensitivity 89.2%, specificity 86.5%	★★-★★★
Marcuello M et al. Cancers, 2019 [67]	FIT+ screening patients, Barcelona, Spain (validation study)	-	59	AA: 74	80	miR-29a-3p, miR-15b-5p, miR-18a-5p, miR-19a-3p, miR-19b-3p, miR-335-5p	Multi-marker (6 miRNAs), CRC: AUC 0.74 (0.65–0.82), sensitivity 81%, specificity 56% Multi-marker (6 miRNAs), AA: AUC 0.80 (0.72–0.87), sensitivity 81%, specificity 63%	★★ - ★★
Zanutto S, et al. Int J Cancer, 2020 [69]	FIT+ screening patients, Milan, Italy(discovery and validation sets)	-	Ext. valid. 33	Ext. valid.AA:181 NAA: 313	Ext. valid. 568	miRNA Taqman array 13 miRNAS selected for validation (of which 4 excluded after hemolysis experiments) plus one candidate from a previous study	Individual markers, CRC: AUC ~0.6 for 5 best miRNAs, AA: AUC range for all miRNAs 0.589–0.608Multi-marker, CRC (hsa-miR-378, hsa-miR-342-3p): AUC 0.604 (0.504–0.704) Multi-marker, AA (hsa-miR-106b-5p, hsa-miR-483-5p, hsa-miR-323a-3p, hsa-miR-335-5p, hsa-miR-186-5p, hsa-miR-342-3p): AUC 0.608 (0.560–0.656)	★★ ★★ ★★★

Abbreviations (not including biomarker names, for which the reader is referred to the original study article): **A**, adenoma; **AA**, advanced adenoma; **AUC,** Area Under the Curve; **BCST**, Bowel Cancer Screening Programme; **BliTz**, Begleitende Evaluierung innovativer Testverfahren zur Darmkrebs-Früherkennun, Germany; **DFTJ,** Dongfeng–Tongji cohort; **EPIC**, European Prospective Investigation into Cancer and Nutrition; **FIT**, fecal immunochemical test; **FOB**, fecal occult blood; **FOBT**, fecal occult blood test; **miRNA**, micro-RNA; **NAA**, non-advanced adenoma; **NSHDS**, North Sweden Health and Disease Study; **PRESEPT**, PRospective Evaluation of SEPTin 9, United States and Germany; **rtPCR**, real-time PCR; **SWHS**; Shanghai Women’s Health Study; **TZL**, Taizhou Longitudinal Study; **USA**, United States of America. * Matched controls. ** Newcastle–Ottawa Quality Assessment Scale for case-control studies, adapted for use for assessment of biomarker studies conducted in a screening setting. For case-control studies: selection (max 4 stars), comparability (max 2 stars), exposure (max 3 stars). For cohort studies: selection (max 4 stars), comparability (max 2 stars), outcome (max 3 stars). *** Cohort design. Total screening colonoscopy cohort: 195 of which 34 completely normal. NOS assessment using the cohort scale. **** Commercially sponsored.

**Table 5:** Studies of other biomarkers analyzed in pre-diagnostic blood samples (collected in prospective cohort or general screening settings) for the purpose of risk prediction or early diagnosis of colorectal cancer.

Reference	Cohort (Design)	Time from Sampling to Diagnosis (Cohort Setting Only)	CRC	Adenoma	Contr./ Cohort	Biomarker/ Platform	Main Findings	Adapted NOS Scale ** Max: Selection = ★★★★ Comp. = ★★ Exp./Outc. = ★★★
**Cohort setting**								
Perttula et al. Cancer Epidemiol Biomarkers Prev, 2016 [80]	EPIC-TURIN (Nested case control)	7.1 years (baseline)	95	-	95 *	Ultra-long Chain Fatty Acids (ULCFA)	Top markers: ULCFAs: 446, 466, 468, 492 and 494 Differences diminished with increasing time to diagnosis	★★ ★★ ★★★
Prizment et al. Cancer Epidemiol Biomarkers Prev, 2016 [81]	ARIC (Cohort)	14.8 years (median follow up)	255	-	12,300	Beta-2-microglobulin (B2M)	HR (4th vs. 1st quartile): 2.21 (95% CI: 1.32–3.70)	★★★ ★★ ★★★
Doherty et al. Sci Rep, 2018 [82]	FINRISK (Nested case control)	10 years (follow up)	40	-	80 *	Plasma N-glycans	Top markers: F(6)A2G2, F(6)A2G2S(6)1 All peaks + age: AUC: 0.65 Sensitivity: 12.5% Specificity: 95%	★★★ ★★ ★★★
Pilling et al. Plos One, 2018 [83]	UK BIOBANK (Cohort)	9 years (follow up)	1327	-	240,477	Red Blood Cell Distribution Width (RDW)	Higher RDW: sHR: 1.92 (95% CI: 1.36 to 2.72)	★★★ ★★ ★★★
Okamura et al. Bmc Endocr Disord, 2020 [79]	NAGALA (Cohort)	4.4 years (median)	116	-	27,921	Triglyceride–glucose index (TyG index)	HR (TyG index): 1.38 (95% CI: 1.0–1.9) AUC: 0.69 Sensitivity: 62% Specificity: 67%	★★ ★★ ★★★
Le Cornet et al. Cancer Res, 2020 [84]	EPIC-HEIDELBERG (Case cohort)	6.7 years (mean)	111	-	465	Immune cell counts (neutrophils, monocytes, and lymphocytes	Top finding: FOXP3+ T-cell counts HR: 1.59 (95% CI: 1.04–2.42)	★★★★ ★★ ★★★

Abbreviations (not including biomarker names, for which the reader is referred to the original study article): **A**, adenoma; **AA**, advanced adenoma; **ARIC**, Atherosclerosis Risk in Communities; **AUC,** Area Under the Curve; **CRC**, Colorectal Cancer; **EPIC**, European Prospective Investigation into Cancer and Nutrition; **FINRISK**, The National FINRISK Study; **HR**, Hazard Ratio; **NAGALA**, NAfld in the Gifu Area, Longitudinal Analysis; **RDW**, Red Blood Cell Distribution Width; **UK BIOBANK**, United Kingdom Biobank. * Matched controls. ** Newcastle–Ottawa Quality Assessment Scale for case-control studies, adapted for use for assessment of biomarker studies conducted in a screening setting. For case-control studies: selection (max 4 stars), comparability (max 2 stars), exposure (max 3 stars). For cohort studies: selection (max 4 stars), comparability (max 2 stars), outcome (max 3 stars).
